# Supplementary material for: The Power of Open Health Data: Impact, Representation, and Knowledge Diffusion
Source: medRxiv. 2026 Mar 25:2026.03.20.26348933. Preprint. [Version 2] doi: 10.64898/2026.03.20.26348933 (PMC13042117; doi:10.64898/2026.03.20.26348933)
Supplement: Supplement 1 [file NIHPP2026.03.20.26348933v2-supplement-1.pdf]

medRxiv preprint doi: https://doi.org/10.64898/2026.03.20.26348933; this version posted March 25, 2026. The copyright holder for this preprint (which was not certified by peer review) is the author/funder, who has granted medRxiv a license to display the preprint in perpetuity.
It is made available under a CC-BY 4.0 International license .

Supplementary Materials

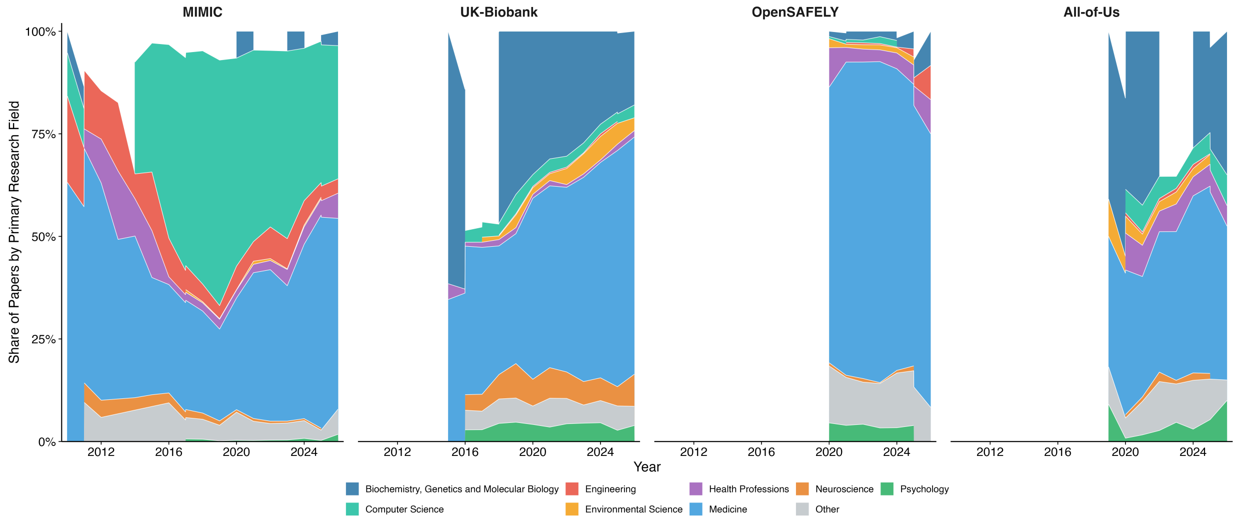

Figure S1: Disciplinary composition of first-degree publications over time by repository. Each color represents a research field as classified by OpenAlex. Only year–repository combinations with ≥10 papers are shown. MIMIC is the only repository whose primary citing field is computer science (43.3%) rather than medicine, reflecting its widespread adoption as a machine learning benchmark. UK Biobank is dominated by biochemistry, genetics, and molecular biology. OpenSAFELY and All of Us remain concentrated within medicine.

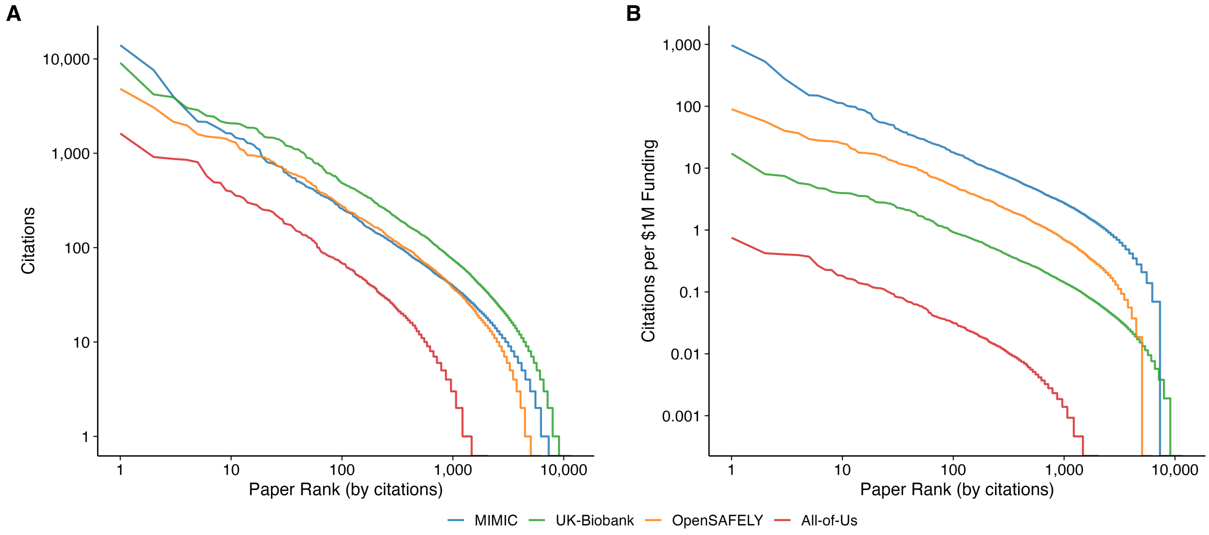

Figure S2: Citation distributions for first-degree publications. Panel A: Raw citation counts by paper rank (log-log scale). Panel B: Funding-normalized citations per $1M by paper rank (log-log scale). MIMIC papers are more highly cited per dollar of funding across the full rank distribution.

medRxiv preprint doi: https://doi.org/10.64898/2026.03.20.26348933; this version posted March 25, 2026. The copyright holder for this preprint (which was not certified by peer review) is the author/funder, who has granted medRxiv a license to display the preprint in perpetuity.
It is made available under a CC-BY 4.0 International license .

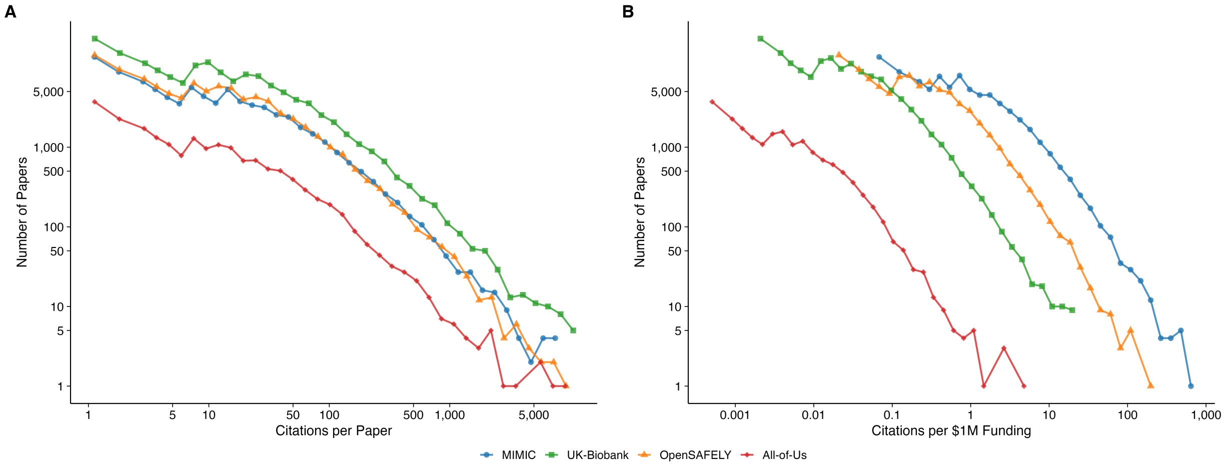

Figure S3: Citation distributions for second-degree publications. Panel A: Raw citation distribution (number of papers at each citation count). Panel B: Funding-normalized citation distribution. The distributions are broadly similar across repositories, consistent with the ∼10× amplification constant.

Table S1: Pairwise chi-square tests comparing MIMIC with each other repository for LMIC representation and gender distribution. All p-values are Bonferroni-corrected ($n = 3$).

Comparison	Measure	χ^2	p (adj)	V	OR (95% CI)
MIMIC vs. UK Biobank	LMIC	5,527.8	<0.001	0.186	2.34 (2.29–2.40)
MIMIC vs. UK Biobank	Gender	792.7	<0.001	0.066	0.74 (0.73–0.76)
MIMIC vs. OpenSAFELY	LMIC	8,156.3	<0.001	0.278	3.53 (3.43–3.63)
MIMIC vs. OpenSAFELY	Gender	1,317.8	<0.001	0.102	0.65 (0.64–0.67)
MIMIC vs. All of Us	LMIC	9,511.7	<0.001	0.378	17.71 (16.45–19.07)
MIMIC vs. All of Us	Gender	884.3	<0.001	0.106	0.61 (0.59–0.63)

OR for LMIC: odds of LMIC authorship in MIMIC relative to comparator (OR >1 = higher in MIMIC). OR for gender: odds of female authorship in MIMIC relative to comparator (OR <1 = lower in MIMIC).

medRxiv preprint doi: https://doi.org/10.64898/2026.03.20.26348933; this version posted March 25, 2026. The copyright holder for this preprint (which was not certified by peer review) is the author/funder, who has granted medRxiv a license to display the preprint in perpetuity.
It is made available under a CC-BY 4.0 International license .

Table S2: Proportion of female authors within HIC and LMIC subgroups, by authorship position. Female representation is higher among LMIC authors than HIC authors in three of the four repositories (MIMIC, UK Biobank, OpenSAFELY); All of Us shows mixed results with small LMIC sample sizes.

		**HIC Female (%)**		**LMIC Female (%)**	
**Repository**	**Position**	*n*	%	*n*	%
MIMIC	First	1,400	30.0	1,310	36.4
	Middle	5,089	29.1	5,081	38.4
	Last	1,109	23.9	1,044	31.9
UK Biobank	First	3,048	40.8	1,384	44.7
	Middle	26,271	37.4	9,582	44.7
	Last	2,145	29.5	926	35.3
OpenSAFELY	First	1,777	42.6	542	46.2
	Middle	16,606	42.2	3,653	46.8
	Last	1,325	34.6	400	37.5
All of Us	First	745	43.1	30	40.0
	Middle	6,500	43.6	281	43.4
	Last	611	37.9	24	48.0

medRxiv preprint doi: https://doi.org/10.64898/2026.03.20.26348933; this version posted March 25, 2026. The copyright holder for this preprint (which was not certified by peer review) is the author/funder, who has granted medRxiv a license to display the preprint in perpetuity.
It is made available under a CC-BY 4.0 International license .

Table S3: Unique author demographics by repository. Authors are deduplicated by OpenAlex author ID within each dataset.

	**MIMIC**	**UK Biobank**	**OpenSAFELY**	**All of Us**
Unique authors	33,573	51,783	44,441	13,238
Female (%)	33.5	41.3	43.7	44.4
LMIC (%)	47.3	30.2	21.8	5.2
Ever first author (n)	6,921	7,278	4,810	1,517
Female (%)	32.8	42.7	43.0	43.8
LMIC (%)	39.2	31.3	21.3	3.8
Ever last author (n)	5,853	5,071	4,289	1,271
Female (%)	28.3	32.8	35.5	39.4
LMIC (%)	40.7	27.0	21.5	3.3

“Ever first/last author” = unique authors who held that position on at least one publication citing the repository. MIMIC’s unique LMIC authorship (47.3%) exceeds its record-level rate (41.8%) because LMIC authors appear on fewer papers on average.

Table S4: Genderize.io gender identification rates (%) by World Bank region and repository. Rates represent the proportion of unique authors for whom Genderize.io returned a gender assignment. South Asia consistently shows the lowest identification rates across repositories.

**Region**	**MIMIC**	**UK Biobank**	**OpenSAFELY**	**All of Us**
	n (%)	n (%)	n (%)	n (%)
North America	8,321 (99.2)	11,975 (99.2)	7,772 (99.4)	8,320 (99.2)
Europe & Central Asia	5,935 (98.9)	16,304 (99.1)	18,300 (98.4)	1,920 (99.5)
East Asia & Pacific	15,135 (98.7)	16,571 (98.9)	5,362 (98.6)	1,124 (99.4)
Middle East & North Africa	741 (99.1)	449 (99.6)	2,002 (99.5)	99 (100.0)
Latin America & Caribbean	492 (99.4)	377 (98.9)	2,234 (99.3)	163 (99.4)
South Asia	1,019 (96.8)	318 (97.8)	1,084 (97.0)	71 (95.8)
Sub-Saharan Africa	92 (97.8)	138 (98.6)	744 (98.8)	103 (99.0)
Unknown/Unclassified	4,881 (98.2)	5,651 (98.6)	6,943 (97.5)	1,438 (99.0)
Total	36,616 (98.7)	51,783 (99.0)	44,441 (98.5)	13,238 (99.2)

Identification rates for South Asia (95.8–97.8%) are systematically lower than other regions, consistent with known limitations of name-based gender inference for South and Southeast Asian names [23].
